# Continue nursing education: an action research study on the implementation of a nursing training program using the Holton Learning Transfer System Inventory

**DOI:** 10.1186/s12909-024-05552-6

**Published:** 2024-06-03

**Authors:** MingYan Shen, ZhiXian Feng

**Affiliations:** 1 School of Nursing, Zhejiang Shuren University, 8 Shuren Road, 310015 Hangzhou, ZheJiang China; 2 Department of Nursing, Shulan (Hangzhou) Hospital, Shulan International Medical College, Zhejiang Shuren University, 310022 Hangzhou, China

**Keywords:** Nursing education, Quality improvement, Action research, Holton Learning Transfer System Inventory

## Abstract

**Objective:**

To address the gap in effective nursing training for quality management, this study aims to implement and assess a nursing training program based on the Holton Learning Transfer System Inventory, utilizing action research to enhance the practicality and effectiveness of training outcomes.

**Methods:**

The study involved the formation of a dedicated training team, with program development informed by an extensive situation analysis and literature review. Key focus areas included motivation to transfer, learning environment, and transfer design. The program was implemented in a structured four-step process: plan, action, observation, reflection.

**Results:**

Over a 11-month period, 22 nurses completed 14 h of theoretical training and 18 h of practical training with a 100% attendance rate and 97.75% satisfaction rate. The nursing team successfully led and completed 22 quality improvement projects, attaining a practical level of application. Quality management implementation difficulties, literature review, current situation analysis, cause analysis, formulation of plans, implementation plans, and report writing showed significant improvement and statistical significance after training.

**Conclusion:**

The study confirms the efficacy of action research guided by Holton’s model in significantly enhancing the capabilities of nursing staff in executing quality improvement projects, thereby improving the overall quality of nursing training. Future research should focus on refining the training program through long-term observation, developing a multidimensional evaluation index system, exploring training experiences qualitatively, and investigating the personality characteristics of nurses to enhance training transfer effects.

## Introduction

The “Medical Quality Management Measures“ [1] and “Accreditation Standards for Tertiary Hospitals (2020 Edition)” [2] both emphasize the importance of using quality management tools in medical institutions to carry out effective quality management [3]. However, there is a notable gap in translating theoretical training into effective, practical application in clinical settings [4]. This gap is further highlighted in the context of healthcare quality management, as evidenced in studies [5] which demonstrate the universality of these challenges across healthcare systems worldwide.

Addressing this issue, contemporary literature calls for innovative and effective training methods that transition from passive knowledge acquisition to active skill application [6]. The Holton Learning Transfer System Inventory [7] provides a framework focusing on key factors such as motivation, learning environment, and transfer design [7–9]. This study aims to implement a nursing training program based on the Holton model, using an action research methodology to bridge the theoretical-practical gap in nursing education.

Quality management training for clinical nurses has predominantly been characterized by short-term theoretical lectures, a format that often fails to foster deep engagement and lasting awareness among nursing personnel [10]. The Quality Indicator Project in Taiwan’s nursing sector, operational for over a decade, demonstrates the effective use of collective intelligence and scientific methodologies to address these challenges [11]. The proposed study responds to the need for training programs that not only impart knowledge but also ensure the practical application of skills in real-world nursing settings, thereby contributing to transformative changes within the healthcare system [12].

In April 2021, the Nursing Education Department of our hospital launched a quality improvement project training program for nurses. The initiation of this study is underpinned by the evident disconnect between theoretical training and the practical challenges nurses face in implementing quality management initiatives, a gap also identified in the work [13]. By exploring the efficacy of the Holton Learning Transfer System Inventory, this study seeks to enhance the practical application of training and significantly contribute to the field of nursing education and quality management in healthcare.

## Developing a nursing training program with the Holton Learning Transfer System Inventory

### Establishing a research team and assigning roles

There are 10 members in the group who serve as both researchers and participants, aiming to investigate training process issues and solutions. The roles within the group are as follows: the deputy dean in charge of nursing is responsible for program review and organizational support, integrating learning transfer principles in different settings [14]; the deputy director of the Nursing Education Department handles the design and implementation of the training program, utilizing double-loop learning for training transfer [15]; the deputy director of the Nursing Department oversees quality control and project evaluation, ensuring integration of evidence-based practices and technology [16] and the deputy director of the Quality Management Office provides methodological guidance. The remaining members consist of 4 faculty members possessing significant university teaching experience and practical expertise in quality control projects, and 2 additional members who are jointly responsible for educational affairs, data collection, and analysis. Additionally, to ensure comprehensive pedagogical guidance in this training, professors specializing in nursing pedagogy have been specifically invited to provide expertise on educational methodology.

### Current situation survey

**Fig. 1:**
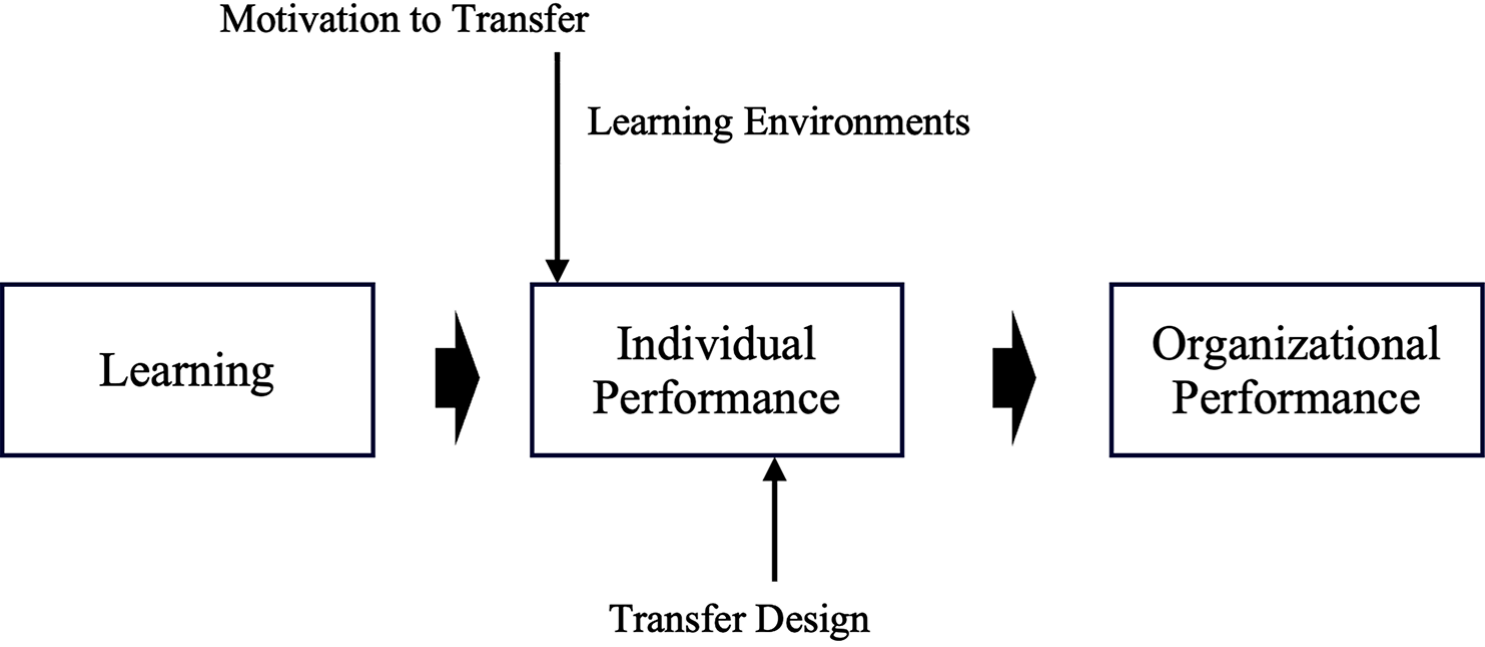
Learning Transfer System Inventory

Based on the Holton Learning Transfer System Inventory (refer to Fig. 1), the appropriate levels of Motivation to Improve Work Through Learning (MTIWL), learning environment, and transfer design are crucial in facilitating changes in individual performance, thereby influencing organizational outcomes [17, 18]. Motivation to Improve Work Through Learning (MTIWL) is closely linked to expectation theory, fairness theory, and goal-setting theory, significantly impacting the positive transfer of training [19]. Learning environment encompasses environmental factors that either hinder or promote the application of learned knowledge in actual work settings [20]. Transfer design, as a pivotal component, includes training program design and organizational planning.

To conduct the survey, the research team retrieved 26 quality improvement reports from the nursing quality information management system, which were generated by nursing units in 2020. A checklist was formulated, and a retrospective evaluation was conducted across eight aspects, namely, team participation, topic selection feasibility, method accuracy, indicator scientificity, program implementation rate, effect maintenance, and promotion and application. Methods employed in the evaluation process included report analysis, on-site tracking, personnel interviews, and data review within the quality information management system [21]. From the perspective of motivation [22], learning environment [23], and transfer design, a total of 14 influencing factors were identified. These factors serve as a reference for designing the training plan and encompass the following aspects: lack of awareness regarding importance, low willingness to participate in training, unclear understanding of individual career development, absence of incentive mechanisms, absence of a scientific training organization model, lack of a training quality management model, inadequate literature retrieval skills and support, insufficient availability of practical training materials and resources, incomplete mastery of post-training methods, lack of cultural construction plans, suboptimal communication methods and venues, weak internal organizational atmosphere, inadequate leadership support, and absence of platforms and mechanisms for promoting and applying learned knowledge.

### Development of the training program using the 4W1H approach

**Fig. 2:**
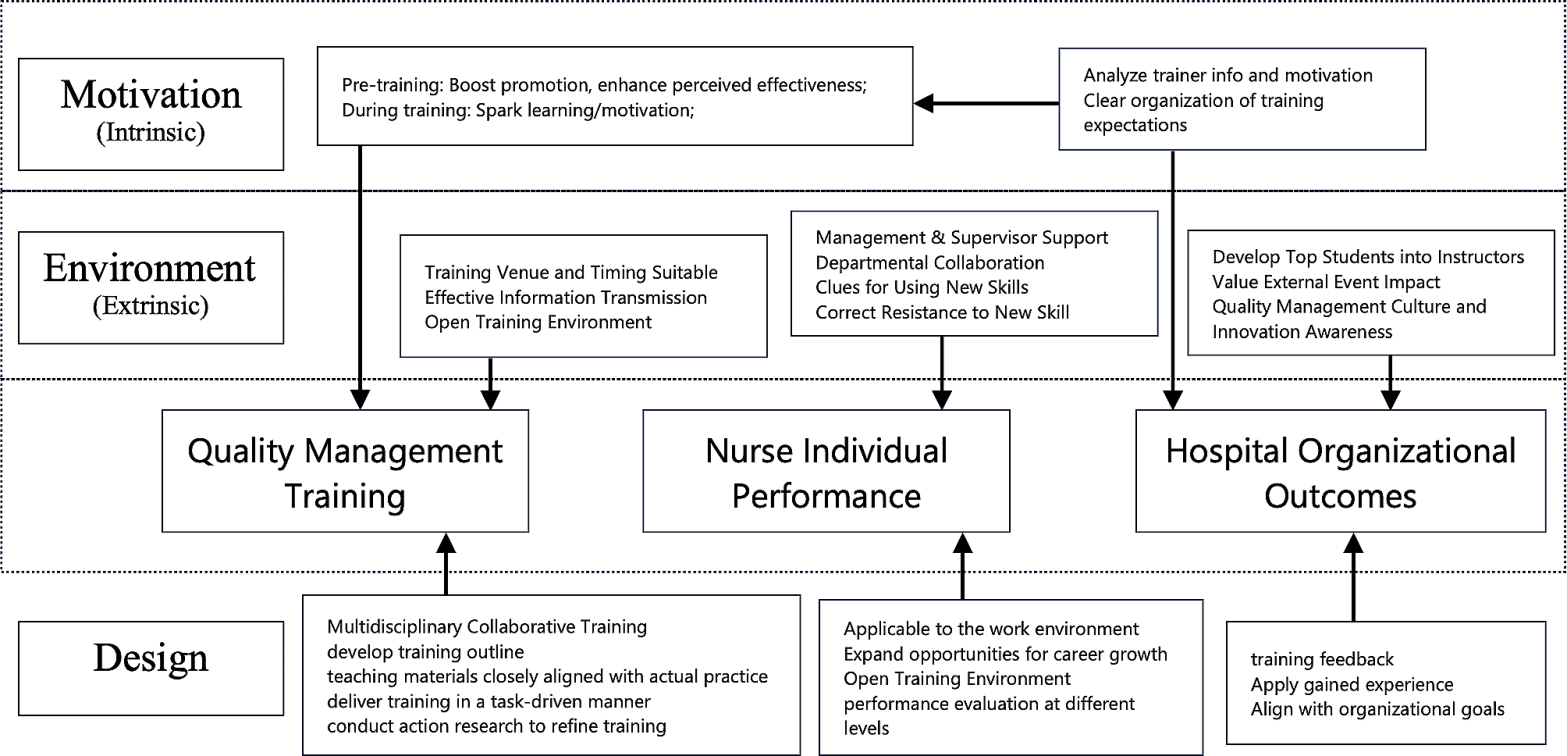
In-house training model from Holton Learning Transfer

Drawing upon Holton’s Learning Transfer System Inventory and the hospital training transfer model diagram, a comprehensive training outline was formulated for the training program [24, 25]. The following components were considered:

(1) Training Participants (Who): The training is open for voluntary registration to individuals with an undergraduate degree or above, specifically targeting head nurses, responsible team leaders, and core members of the hospital-level nursing quality control team. Former members who have participated in quality improvement projects such as Plan-Do-Check-Act Circle (PDCA) or Quality control circle (QCC) are also eligible.

(2) Training Objectives (Why): At the individual level, the objectives include enhancing the understanding of quality management concepts, improving the cognitive level and application abilities of project improvement methods, and acquiring the necessary skills for nursing quality improvement project. At the team level, the aim is to enhance effective communication among team members and elevate the overall quality of communication. Moreover, the training seeks to facilitate collaborative efforts in improving the existing nursing quality management system and processes. At the operational level, participants are expected to gain the competence to design, implement, and manage nursing quality improvement project initiatives. Following the training, participants will lead and successfully complete a nursing quality improvement project, which will undergo a rigorous audit.

(3) Training Duration (When): The training program spans a duration of 11 months.

(4) Training Content (What): The program consists of 14 h of theoretical courses and 18 h of practical training sessions, as detailed in Table 1.

(5) Quality Management Approach (How): To ensure quality throughout the training process, two team members are assigned to monitor the entire training journey. This encompasses evaluating whether quality awareness education, quality management knowledge, and professional skills training are adequately covered. Additionally, attention is given to participants’ learning motivation, the emphasis placed on active participation in training methods, support from hospital management and relevant departments, as well as participants’ satisfaction and assessment results. Please refer to Fig. 2 for a visual representation.

**Table 1:** Training program table

Course Nature	Title	Content	Class Hours	Cycle
Theory	Overview of Nursing Improvement Projects	What is a nursing improvement project; reasons for implementing nursing improvement projects; steps involved in nursing improvement activities; key factors for successful implementation of nursing improvement projects; similarities and differences between nursing projects and other quality management initiatives	1	First
Theory	Methods for Selecting Project Topics	Defining project problems; sources for project topics; how to determine project topics; considerations for writing project topics; discussion on quality management indicators	2	First
Theory	Analysis of Common Tools in Nursing Improvement Projects	Checklists, scatter plots, stratification, histograms, Pareto charts, fishbone diagrams, control charts	3	First
Theory	Methods for Literature Retrieval	Considerations for literature retrieval in project work; usage of commonly used Chinese and English databases	3	First
Theory	Development and Progression of Project Improvement Plans	Current situation analysis; goal setting; formulation of solution plans; implementation of solution plans; evaluation of results; obstacles and facilitating factors during the process	3	First
Theory	Utilizing Leadership to Drive Project Improvement Practices	Defining leadership; common leadership models; role of leadership in hospital quality improvement; visible leadership	1	First
Theory	Writing Nursing Project Reports	Considerations for writing nursing project reports	1	First
Practical Training	Group Presentation: Project Topic Selection	8 min/project presentation, followed by feedback from experts and other participants	3	Second
Practical Training	Group Presentation: Current Situation Analysis and Goal Setting	8 min/project presentation, followed by feedback from experts and other participants	3	Second
Practical Training	Group Presentation: Current Situation Analysis and Goal Setting	8 min/project presentation, followed by feedback from experts and other participants	3	Second
Practical Training	Group Presentation: Implementation Phase - Obstacles and Facilitating Factors	8 min/project presentation, followed by feedback from experts and other participants	3	Second
Practical Training	On-site Tracking of Implementation Process	Experts provide on-site guidance and tracking for the implementation of project initiatives	3	Second
Practical Training	Group Presentation: Evaluation Phase - Project Improvement Effectiveness and Case Report Writing	8 min/project presentation, followed by feedback from experts and other participants	3	Third

## Implementation of the nursing project training program using the action research method

### The first cycle (April 2021)

In the initial cycle, a total of 22 nurses were included as training participants after a self-registration process and qualification review. The criteria used to select these participants, elaborated in Section Development of the training program using the 4W1H approach, ‘Development of the Training Program,’ were meticulously crafted to capture a broad spectrum of experience, expertise, and functional roles within our hospital’s nursing staff. The primary focus was to investigate their learning motivation. The cycle comprised the following key activities:

(1) Training Objectives: The focus was on understanding the learning motivation of the participating nurses.

(2) Theoretical Training Sessions: A total of 7 theoretical training sessions, spanning 14 class hours, were completed. The contents covered various aspects, including an overview of nursing quality improvement projects, methods for selecting project topics, common tools used in nursing quality improvement projects, effective leadership strategies to promote project practices, literature retrieval and evaluation methods, formulation and promotion of project plans, and writing project reports. Detailed course information, including the title, content, and class hours, is listed in Table 1. At the end of each training session, a course satisfaction survey was conducted.

(3) Assessment and Reporting: Following the completion of the 7 training sessions, a theoretical assessment on quality management knowledge was conducted. Additionally, nurses were organized to present their plans for special projects to be carried out during the training. Several issues were identified during this cycle:

- Incomplete Literature Review Skills: Compared to other quality control tools, nursing quality improvement project places more emphasis on the scientific construction of project plans. The theoretical evaluation and interviews with nurses highlighted the incomplete and challenging nature of their literature review skills.
- Insufficient Leadership: Among the participants, 6 individuals were not head nurses, which resulted in a lack of adequate leadership for their respective projects.
- Learning environment and Support: The learning environment, as well as the support from hospital management and relevant departments, needed to be strengthened.

### Second cycle (may-october 2021)

In response to the issues identified during the first cycle, our approach in the second cycle was both corrective and adaptive, focusing on immediate issues while also setting the stage for addressing any emerging challenges. The team members actively implemented improvements during the second cycle. The key actions taken were as follows:

(1) Establishing an Enabling Organizational Environment: The quality management department took the lead, and multiple departments collaborated in conducting the “Hospital Safety and Quality Red May” activity. This initiative aimed to enhance the overall quality improvement atmosphere within the hospital. Themed articles were also shared through the hospital’s WeChat public account.

(2) Salon-style Training Format: The training sessions were conducted in the form of salons, held in a meeting room specifically prepared for this purpose. The room was arranged with a round table, warm yellow lighting, green plants, and a coffee bar, creating a conducive environment for free, democratic, and equal communication among the participants. The salon topics included revising project topic selection, conducting current situation investigations, facilitating communication and guidance for literature reviews, formulating improvement plans, implementing those plans, and writing project reports. After the projects were presented, quality management experts provided comments and analysis, promoting the transformation of training outcomes from mere memory and understanding to higher-level abilities such as application, analysis, and creativity.

(3) Continuous Support Services: Various support services were provided to ensure ongoing assistance. This included assigning nursing postgraduates to aid in literature retrieval and evaluation. Project team members also provided on-site guidance and support, actively engaging in the project improvement process to facilitate training transfer.

(4) Emphasis on Spiritual Encouragement: The Vice President of Nursing Department actively participated in the salons and provided feedback on each occasion. Moreover, the President of the hospital consistently commended the training efforts during the weekly hospital meetings.

#### Issues identified in this cycle

(1) Inconsistent Ability to Write Project Documents: The proficiency in writing project documents for project improvement varied among participants, and there was a lack of standardized evaluation criteria. This issue had the potential to impact the quality of project dissemination.

(2) Lack of Clarity Regarding the Platform and Mechanism for Training Result Transfer: The platform and mechanisms for transferring training results were not clearly defined, posing a challenge in effectively sharing and disseminating the outcomes of the training.

### The third cycle (November 2021-march 2022)

#### During the third cycle, the following initiatives were undertaken

(1) Utilizing the “Reporting Standards for Quality Improvement Research (SQUIRE)”, as issued by the US Health Care Promotion Research, to provide guidance for students in writing nursing project improvement reports.

(2) Organizing a hospital-level nursing quality improvement project report meeting to acknowledge and commend outstanding projects.

(3) Compiling the “Compilation of Nursing Quality Improvement Projects” for dissemination and exchange among nurses both within and outside the hospital.

(4) Addressing the issue of inadequate management of indicator monitoring data, a hospital-level quality index management platform was developed. The main evaluation data from the 22 projects were entered into this platform, allowing for continuous monitoring and timely intervention.

## Effect evaluation

To assess the efficacy of the training, a diverse set of evaluation metrics, encompassing both outcome and process measures [26]. These measures can be structured around the four-level training evaluation framework proposed by Donald Kirkpatrick [27].

### Process evaluation

#### Evaluation method

To assess the commitment and support within the organization, the process evaluation involved recording the proportion of nurses’ classroom participation time and the presence of leaders during each training session. Additionally, a satisfaction survey was conducted after the training to assess various aspects such as venue layout, time arrangement, training methods, lecturer professionalism, content practicality, and interaction. On-site recycling statistics were also collected for project evaluation purposes.

#### Evaluation results the results of the process evaluation are as follows

- Nurse training participation rate: 100%.
- Training satisfaction rate (average): 97.75%.
- Proportion of nurses’ participation time in theoretical training sessions (average): 36.88%.
- Proportion of nurses’ participation time in salon training sessions (average): 74.23%.
- Attendance rate of school-level leaders: 100%.

### Results evaluation

#### Assessment of theoretical knowledge of quality management

To evaluate the effectiveness in enhancing the trainees’ theoretical knowledge of quality management, the research team conducted assessments before the training, after the first round of implementation, and after the third round of implementation. Assessments to evaluate the effectiveness of the training program were conducted immediately following the first round of implementation, and after the third round of implementation. This dual-timing approach was designed to evaluate both the immediate impact of the training and its sustained effects over time, addressing potential influences of memory decay on the study results. The assessment consisted of a 60-minute examination with different question types, including 30 multiple-choice questions (2 points each), 2 short-answer questions (10 points each), and 1 comprehensive analysis question (20 points). The maximum score achievable was 100 points.

The assessment results are as follows:

- Before training (average): 75.05 points.
- After the first round of implementation (average): 82.18 points.
- After the third round of implementation (average): 90.82 points.

**Table 2:** Pre- and Post-training project implementation challenges assessment results

Item	Implementation Challenges Assessment(Points)	t	*P*
Literature Review	1.86 ± 1.70	5.146	< 0.001
Current situation	1.64 ± 1.59	4.827	< 0.001
Plan formulation	0.91 ± 1.31	3.265	0.004
Goal setting	0.73 ± 1.16	2.935	0.008
Cause Analysis	1.86 ± 1.58	5.524	< 0.001
Plan Development	1.32 ± 1.39	4.437	< 0.001
Plan Implementation	1.73 ± 1.03	7.851	< 0.001
Effect Evaluation	0.91 ± 1.38	3.097	0.005
Report Writing	2.68 ± 1.43	8.814	< 0.001

#### Assessment of difficulty in quality management project implementation

To assess the difficulty of implementing quality management projects, the trainees completed the “Quality Management Project Implementation Difficulty Assessment Form” before and after the training. They self-evaluated 10 aspects using a 5-point scale, with 5 indicating the most difficult and 1 indicating no difficulty. The evaluation results before and after implementation are presented in Table 2.

Statistically significant differences were found in the following items: literature review, current situation analysis, cause analysis, plan formulation, implementation plan, and report writing. This indicates that the training significantly enhanced the nurses’ confidence and ability to tackle practical challenges.

#### Evaluation of transfer effect

To assess how effectively the training translated into practical applications. The implementation of the 22 quality improvement projects was evaluated using the application hierarchy analysis table. The specific results are presented in Table 3.

In addition, the “Nursing Project Guidance Manual” and “Compilation of Nursing Project Improvement Projects” were compiled and distributed to the hospital’s management staff, nurses, and four collaborating hospitals, receiving positive feedback. The lecture titled “Improving Nurses’ Project Improvement Ability Based on the Training Transfer Theory Model” shared experiences with colleagues both within and outside the province in national and provincial teaching sessions in 2022. Furthermore, four papers were published on the subject.

**Table 3:** Application levels for 22 quality improvement projects

Category	State	Characteristic	Description	Number of Items	Percentage(%)
Application	6	Update	Innovatively assess the quality of applications and identify areas for improvement	2	8.70
	5	Assimilation	Collaborate effectively with colleagues to create a stronger collective impact	7	30.43
	4b	Refine	Adapt application methods based on specific contextual changes	5	21.74
	4a	Routine	Consistently adopt established and fixed application models	8	37.78
	3	Mechanical	Apply mechanically without modification	0	0
Non-application	2	Preparation	Final preparation for initial application	0	0
	1	Orientation	Have application intentions, understand individual and resource requirements for application	0	0
	0	None	No application, current actions are completely unrelated to learning	0	0

## Discussion

### The effectiveness of the training program based on the Holton Learning transfer System Inventory

The level of refined management in hospitals is closely tied to the quality management awareness and skills of frontline medical staff. Quality management training plays a crucial role in improving patient safety management and fostering a culture of quality and safety. Continuous quality improvement is an integral part of nursing management, ensuring that patients receive high-quality and safe nursing care. Compared to the focus of existing literature on the individual performance improvements following nursing training programs [28–30], our study expands the evaluation framework to include organizational performance metrics. Our research underscores a significantly higher level of organizational engagement as evidenced by the 100% attendance rate of school-level leaders. The publication of four papers related to this study highlights not only individual performance achievements but also significantly broadens the hospital organization’s impact on quality management, leading to meaningful organizational outcomes.

Moreover, our initiative to incorporate indicators of quality projects into a hospital-level evaluation index system post-training signifies a pivotal move towards integrating quality improvement practices into the very fabric of organizational operations. In training programs, it is essential not only to achieve near-transfer, but also to ensure that nurses continuously apply the acquired management skills to their clinical work, thereby enhancing quality, developing their professional value, and improving organizational performance. The Holton learning Transfer System Inventory provides valuable guidance on how to implement training programs and evaluate their training effect.

This study adopts the training transfer model as a framework to explore the mechanisms of “how training works” rather than simply assessing “whether training works [31].” By examining factors such as Motivation to Improve Work Through Learning (MTIWL), learning environment, and transfer design, the current situation is analyzed, underlying reasons are identified, and relevant literature is reviewed to develop and implement training programs based on the results of a needs survey. While individual transfer motivation originates from within the individual, it is influenced by the transfer atmosphere and design. By revising the nurse promotion system and performance management system and aligning them with career development, nurses’ motivation to participate and engage in active learning has significantly increased [32]. At the learning environment level, enhancing the training effect involves improving factors such as stimulation and response that correspond to the actual work environment [33]. This project has garnered attention and support from hospital-level leaders, particularly the nursing dean who regularly visits the training site to provide guidance, which serves as invaluable recognition. Timely publicity and recognition of exemplary project improvement initiatives have also increased awareness and understanding of project knowledge among doctors and nurses, fostering a stronger quality improvement atmosphere within the team.

Transfer design, the most critical component for systematic learning and mastery of quality management tools, is achieved through theoretical lectures, salon exchanges, and project-based training. These approaches allow nurses to gain hands-on experience in project improvement under the guidance of instructors. Throughout the project, nurses connect project management knowledge and skills with practical application, enabling personal growth and organizational development through problem-solving in real work scenarios. Finally, a comprehensive evaluation of the training program was conducted, including assessments of theoretical knowledge, perception of management challenges, and project quality. The results showed high satisfaction among nurses, with a satisfaction rate of 97.75%. The proportion of nurses’ participation time in theoretical and practical training classes was 36.88% and 74.23%, respectively. The average score for theoretical knowledge of quality management increased from 75.05 to 90.82. There was also a significant improvement in the evaluation of the implementation difficulties of quality management projects. Moreover, 22 nurses successfully led the completion of one project improvement project, with six projects focusing on preventing the COVID-19 pandemic, demonstrating valuable crisis response practices.

### Action research helps to ensure the quality of organizational management of training

Well-organized training is the basis for ensuring the scientific and standardized development of nursing project improvement activities. According to the survey results of the current situation, there is a lot of room for improvement in the training quality; since it is the first time to apply the Holton training transfer model to the improvement training process of nurses in the hospital, in order to allow the nurses to have sufficient time to implement and evaluate the improvement project, the total training time Set at 11 months, a strong methodology is required to ensure training management during this period. Action research is a research method that closely combines research with solving practical problems in work. It is a research method aimed at solving practical problems through self-reflective exploration in realistic situations, emphasizing the participation of researchers and researchees. Practice, find problems in practice, and adjust the plan in a timely manner. According to the implementation of the first round, it was found that nurses had insufficient literature review skills, insufficient leadership, and lack of support from hospital management and related departments [32]. In the second round, the training courses were carried out in the form of salons. The project team members went deep into the project to improve on-site guidance, arranged graduate students to assist in document retrieval and evaluation, and promoted the transfer of training; the “Hospital Safety and Quality Red May” activity was carried out, and the vice president of nursing Regularly participate in the salon and make comments. The problems exposed after this round of implementation are the low quality of the project improvement project document, and the unclear platform and mechanism for the transfer of training results. In the third round, the “Reporting Standards for Quality Improvement Research (SQUIRE)” was used to standardize the writing of the report [33], and the “Compilation of Nursing Project Improvement Projects” was completed, and the main evaluation data of 22 projects were entered into the hospital-level quality index management platform for continuous monitoring and intervention. As of May 2022, the effect maintenance data of each project has reached the target value. It can not only produce useful improvement projects, but also help to promote the dissemination and penetration of quality awareness.

### Future research directions

Drawing on the Holton training evaluation model, this study implemented nurse quality improvement project training using action research methodology, resulting in a successful exploration practice, and achieving positive transfer effects. To further advance this research area, the following future research directions are recommended:

1. Summarize the experiences gained from this action research training and continue to refine and enhance the training program. Through ongoing practice, reflection, and refinement in subsequent training sessions, long-term observation of the transfer effects can be conducted to establish an effective experiential model that can serve as a reference for future initiatives.
2. Develop a multidimensional evaluation index system for assessing transfer effects. A comprehensive framework that captures various dimensions of transfer, such as knowledge application, skill utilization, and behavior change, should be established. This will enable a more holistic and accurate assessment of the training program’s impact on the participants and the organization.
3. Conduct qualitative research to explore the training experiences of nurses. By gathering in-depth insights through interviews or focus group discussions, a deeper understanding of the nurses’ perceptions, challenges, and facilitators of training transfer can be obtained. This qualitative exploration will provide valuable information to further refine and tailor the training program to meet the specific needs and preferences of the nurses.
4. Investigate the personality characteristics of nurses who actively engage in training transfer and consider developing them as internal trainers. By identifying and cultivating nurses with a proactive attitude and a strong inclination towards knowledge application and skill development, the organization can enhance employee participation and initiative. These internal trainers can play a crucial role in motivating their colleagues and driving the transfer of training outcomes into daily practice.

By pursuing these future research directions, the field of healthcare and nursing care can continue to advance in optimizing training programs, enhancing transfer effects, and ultimately improving the quality of care and patient outcomes.

## Limitations

The research was conducted with a cohort of 22 nurses and a 10-member research team from Grade 3, Class A hospitals in China Southeast. This specific composition and the relatively small sample size may affect the generalizability of our findings. The experiences and outcomes observed in this study might not fully encapsulate the diverse challenges and environments encountered by nursing professionals in varying healthcare settings. The significant improvements noted in the capabilities of the participating nursing staff underscore the potential impact of the training program. However, the study’s focus on a specific demographic—nurses from high-grade hospitals in a developed urban center—may limit the external validity of the findings.

## Conclusions

This study affirms the efficacy of the Holton Learning Transfer System Inventory-based training program, coupled with action research, in significantly advancing nursing quality management practices. The strategic incorporation of motivation to improve work through learning, an enriched learning environment, and thoughtful transfer design significantly boosted the nurses’ engagement, knowledge acquisition, and practical application of quality management tools in their clinical work.

It highlights the importance of continuous learning, organizational support, and methodological flexibility in achieving sustainable improvements in healthcare quality and safety. Future endeavors should aim to expand the scope of this training model to diverse nursing contexts and evaluate its long-term impact on organizational performance and patient care outcomes.

## Data Availability

The datasets generated and/or analyzed during the current study are not publicly available due to hospital policy but are available from the corresponding author on reasonable request.

## Declarations

Prior to participation, all subjects (or their legal guardians) were informed about the nature, objectives, potential benefits, and risks of the study. Written informed consent was obtained from all subjects and/or their legal guardians. All data were collected and processed maintaining strict confidentiality and anonymity, safeguarding the privacy and rights of all participants.
